# Characteristics of Clinical Studies Used for US Food and Drug Administration Supplemental Indication Approvals of Drugs and Biologics, 2017 to 2019

**DOI:** 10.1001/jamanetworkopen.2021.13224

**Published:** 2021-06-10

**Authors:** Meera Dhodapkar, Audrey D. Zhang, Jeremy Puthumana, Nicholas S. Downing, Nilay D. Shah, Joseph S. Ross

**Affiliations:** 1 Yale School of Medicine, Yale University, New Haven, Connecticut; 2 Department of Medicine, Duke University School of Medicine, Durham, North Carolina; 3 Bain Capital Life Sciences, Boston, Massachusetts; 4 Division of Health Care Policy and Research, Robert D. and Patricia E. Kern Center for the Science of Health Care Delivery, Mayo Clinic, Rochester, Minnesota; 5 Section of General Medicine and the National Clinical Scholars Program, Department of Internal Medicine, Yale University School of Medicine, New Haven, Connecticut; 6 Department of Health Policy and Management, Yale School of Public Health, New Haven, Connecticut; 7 Center for Outcomes Research and Evaluation, Yale-New Haven Health System, New Haven, Connecticut

## Abstract

**Importance:**

After US Food and Drug Administration (FDA) approval of a new drug, sponsors can submit additional clinical data to obtain supplemental approval for use for new indications.

**Objective:**

To characterize pivotal trials supporting recent supplemental new indication approvals of drugs and biologics by the FDA and to compare them with pivotal trials that supported these therapeutics’ original indication approvals.

**Design, Setting, and Participants:**

This is a cross-sectional study characterizing pivotal trials supporting supplemental indication approvals by the FDA between 2017 and 2019 and pivotal trials that supported these therapeutics’ original indication approvals. Data analysis was performed from August to October 2020.

**Main Outcomes and Measures:**

Number and design of pivotal trials supporting both supplemental and original indication approvals.

**Results:**

From 2017 to 2019, the FDA approved 146 supplemental indications for 107 therapeutics on the basis of 181 pivotal efficacy trials. The median (interquartile range) number of trials per supplemental indication was 1 (1-1). Most trials used either placebo (77 trials [42.5%; 95% CI, 35.6%-49.8%]) or active comparators (65 trials [35.9%; 95% CI, 29.3%-43.1%]), and most of these multigroup trials were randomized (141 trials [99.3%; 95% CI, 96.0%-100.0%]) and double-blinded (106 trials [74.5%; 95% CI, 66.6%-81.0%]); 80 trials (44.2%; 95 CI, 37.2%-51.5%) used clinical outcomes as the primary efficacy end point. There was no difference between oncology therapies and those approved for other therapeutic areas to have supplemental indication approvals be based on at least 2 pivotal trials (11.5% vs 20.6%; difference, 9.1%; 95% CI, 2.9%-21.0%; *P* = .10). Similarly, there was no difference in use of randomization (98.3% vs 100.0%; difference, 1.7%; 95% CI, 1.6%-5.0%; *P* = .43) among multigroup trials, although these trials were less likely to be double-blinded (50.8% vs 92.3%; difference, 41.5%; 95% CI, 27.4%-55.5%; *P* < .001); overall, these trials were less likely to use either placebo or active comparators (64.9% vs 86.7%; difference, 21.8% 95% CI, 9.8%-33.9%; *P* < .001) or to use clinical outcomes as their primary efficacy end point (27.5% vs 61.1%; difference, 33.6%; 95% CI, 14.1%-40.9%; *P* < .001) and were longer (median [interquartile range], 17 [6-48] weeks vs 95 [39-146] weeks). Original approvals were more likely than supplemental indication approvals to be based on at least 2 pivotal trials (44.0% [95% CI, 33.7%-42.6%] vs 15.8% [95% CI, 10.7%-22.5%]; difference, 28.2%; 95% CI, 17.6%-39.6%; *P* < .001) and less likely to be supported by at least 1 trial of 12 months’ duration (27.6% [95% CI, 17.9%-35.0%] vs 54.8% [95% CI, 46.7%-62.6%]; difference, 27.2%; 95% CI, 14.5%-37.8%; *P* < .001). Pivotal trial designs were otherwise not significantly different.

**Conclusions and Relevance:**

These findings suggest that the number and design of the pivotal trials supporting supplemental indication approvals by the FDA varied across therapeutic areas, with the strength of evidence for cancer indications weaker than that for other indications. There was little difference in the design characteristics of the pivotal trials supporting supplemental indication and original approvals.

## Key Points

**Question:**

What is the strength of evidence supporting supplemental new indication approvals for drugs and biologics and how does it compare with the evidence that supported their original approval?

**Findings:**

In this cross-sectional study of 107 therapeutics approved for 146 supplemental indications by the US Food and Drug Administration between 2017 and 2019, supplemental approvals for oncology drugs were based on fewer pivotal efficacy trials with less rigorous designs than supplemental approvals for other therapeutic areas. Supplemental approvals were based on fewer pivotal trials than their original indication approvals, but their designs were similar.

**Meaning:**

These findings suggest that there was little difference in the evidence supporting supplemental and original indication approvals, but the number and design of pivotal trials supporting supplemental indication approvals varied according to therapeutic area.

## Teaser

This cross-sectional study seeks to characterize pivotal trials supporting recent supplemental new indication approvals of drugs and biologics by the US Food and Drug Administration (FDA) and compares them with pivotal trials that supported these therapeutics’ original indication approvals.

## Introduction

To receive US Food and Drug Administration (FDA) approval for a new small-molecule or biologic drug, sponsors must submit “adequate and well controlled investigations”^1^ to demonstrate the drug’s efficacy and safety. Although the FDA suggests that sponsors submit at least 2 trials demonstrating efficacy, also known as pivotal efficacy trials, the number and characteristics of pivotal efficacy trials supporting new drug and biologic approvals vary widely.^2,3^ For example, approximately one-third of new approvals are based on a single pivotal trial, whereas nearly one-half are based on trials solely focused on surrogate markers of disease, as opposed to clinical outcomes.^2^ This flexibility highlights the FDA’s ability to adapt its standards to the clinical context and use of the drug or biologic under consideration.^4^

Less is known about the clinical trial evidence that supports supplemental indication approvals by the FDA, which are required when sponsors intend to add use of the originally approved drug for new clinical indications or in specific subpopulations to the labeling. Supplemental indication approvals by the FDA for already marketed drugs occur in frequencies similar to those for approvals for novel therapeutics. For instance, in 2014, the FDA approved 40 new supplemental indications compared with 44 new drug approvals.^5^ Supplemental new indication approvals are particularly common among oncology therapies, such as the anti-programmed death–1 biologic pembrolizumab (Keytruda), which was originally approved for the treatment of advanced melanoma in 2014 and has since been approved for 19 additional clinical indications.^6^ Moreover, it is critical that both new and supplemental indication approvals are based on strong evidence, because certain therapeutics may be prescribed more often for their supplemental indications than for their initially approved indication.^7^ Supplemental indication approval by the FDA is also critical for ensuring insurance coverage, especially for more expensive biological therapies and those used for oncology, where effective treatment options may be more limited.^8^

There have been few studies characterizing the clinical trial evidence that supported supplemental indication approvals by the FDA. One study^9^ broadly examined nearly 300 supplemental indication approvals between 2005 and 2014 and found that approximately one-third used active comparator groups and one-third used clinical outcomes as primary end points. Another study^10^ examined new and supplemental indication approvals, as well as off-label indications included in a Medicare-referenced compendium in oncology, from 2005 to 2012 and found the number and characteristics of supporting pivotal trials to be similar. However, whether recent supplemental indication approvals differ from original approvals more broadly and whether the evidentiary standards required for a supplemental indication approval for oncology therapeutics differ from those required for other therapeutics remain unknown. This information is particularly important in the context of ongoing efforts to increasingly leverage observational studies, as opposed to clinical trials, to support regulatory evaluations and supplemental indication approvals, including federal legislation like the 21st Century Cures Act.

Accordingly, we reviewed all supplemental new drug applications (sNDAs) and supplemental biologics license applications (sBLAs) approved by the FDA for new clinical indications from 2017 to 2019 to describe the characteristics of the pivotal efficacy clinical trials supporting their original indications and supplemental indications. We expect our findings will inform policy makers and clinician decision-making, particularly our understanding of the clinical evidentiary standards for supplemental new indication approvals.

## Methods

This study was prepared in accordance with the Strengthening the Reporting of Observational Studies in Epidemiology (STROBE) reporting guideline for cross-sectional studies. This study did not require institutional review board approval or informed consent because it was based on publicly available information and involved no patient records, in accordance with 45 CFR §46.

### Sample Construction

One study author (M.D.) identified all new drugs and biologics approved for supplemental indications between January 2017 and December 2019 using the Drugs@FDA database. We limited our sample to sNDAs and sBLAs approved by the FDA for new clinical indications, identified through full-text review of sNDA and sBLA approval letters; corresponding FDA approval letters and printed labels were obtained as they are hyperlinked in the Drugs@FDA database. Duplicate records were removed, and sNDAs and sBLAs for contrast agents, nontherapeutic agents, and generics and biosimilars were excluded. Furthermore, we excluded any sBLAs that were determined to be (original) BLAs and removed from FDA’s Orange Book on March 23, 2020, under the Biologics Price Competition and Innovation Act of 2009. Next, for each therapeutic for which an sNDA or sBLA was identified and included in our study, we identified the original indication NDA or BLA approved by the FDA, along with the pivotal trials supporting the FDA’s approval for their original indication using previously described methods.^2,3^

### Supplemental Indication and Regulatory Characteristics

New indication approvals and original indication approvals were characterized by agent type, therapeutic area, and special regulatory status. For each approval, we determined whether the approved therapeutic was a small molecule or biologic from the letter and label. Treatments were characterized as short-term if the therapeutics were expected to be used for less than 1 month, as intermediate if they were expected to be used for 1 month to 2 years, and long-term if they were expected to be used for more than 2 years. Therapeutic area was classified for each approval was based on the World Health Organization Anatomical Therapeutic Classification System.^11^ Using publicly available information on the FDA website, we determined whether each indication in our sample was evaluated under a special regulatory program such as Priority Review,^12^ Accelerated Approval,^13^ Fast Track^14^ for (NDAs and BLAs only), or Breakthrough Therapy.^15^

### Trial Identification

To identify the pivotal clinical trials supporting the sNDAs and sBLAs, we reviewed the drug labels updated by the FDA to correspond to the specific new indication approval, which typically reference the clinical trial using the ClinicalTrials.gov registration number (ie, National Clinical Trials identifier). Generally, those studies considered pivotal are labeled as such in the Clinical Studies section of the printed label. Furthermore, if not directly stated as pivotal, efficacy for an indication is stated to be based on the evidence from a trial or number of trials. If an approval was based on the results of more than 1 trial, all these trials were included in our study. To identify pivotal trials not directly referenced in the label using a National Clinical Trials number, general study information (eg, number of study participants, primary efficacy end point, masking, and randomization) was cross-referenced with study information from ClinicalTrials.gov to identify the relevant National Clinical Trials number.

### Pivotal Trial Characterization

For all pivotal trials supporting NDA, sNDA, BLA, and sBLA approval by the FDA, 1 study author (M.D.) extracted the following information from ClinicalTrials.gov if not available from or previously collected as part of prior research from FDA action packages, including approval letters and review documents: randomization, double-blinding, comparator, study end point, agent type, total number of participants, and number of patients in intervention group.^2,3^ For each trial included in our sample, we determined its use of randomization and double-blinding and characterized the comparator group as active, placebo, or none. Next, the primary trial end point was identified and was designated into 1 of 3 categories—clinical outcome, clinical scale, or surrogate marker—by 1 study author (M.D.) and was reviewed by another study author (J.S.R.); any conflicts were resolved by consensus. In brief, surrogate markers (eg, hemoglobin A_1c_) are metrics expected to be associated with patient outcomes, where clinical outcomes (eg, death) measure patient outcomes. Importantly, if at least 1 primary trial end point was categorized as a clinical outcome or clinical scale, the trial would be categorized as such even if another primary trial end point was a surrogate marker. The number of participants, intervention model, and number of patients in the intervention group were also extracted. Trial duration was extracted from study descriptions using methods previously described.^2,3^ For time-associated end points, duration was defined as the date of measurement of the primary end point. For event-associated end points, duration was determined as the median follow-up time for participants or the weighted average of the median follow-up time in cases in which it differed between study groups.

### Statistical Analysis

We used descriptive statistics to examine differences in trial characteristics and indication characteristics. Frequencies of use of randomization and double-blinding among the trials in our sample were calculated excluding single-group studies. We next used χ^2^ and Wilcoxon tests where appropriate to examine differences between supplemental indication approvals for therapeutics in our sample at multiple levels, including agent type, special regulatory pathway, and therapeutic area, all of which were defined before data collection. Analyses were performed using R statistical software version 3.4.3 (R Project for Statistical Computing), JMP statistical software version 15.0.0 (SAS Institute), Python statistical software version 3.8 (Python), and Excel spreadsheet software version 16.16.27 (Microsoft). All statistical tests were 2-tailed and used a type I error rate of .01 to account for multiple comparisons. Data analysis was performed from August to October 2020.

## Results

Between January 2017 and December 2019, the FDA approved 3721 new sNDA and sBLAs (3200 sNDAs and 521 sBLAs), among which were 146 supplemental indication approvals for a new clinical indication, for a total of 107 therapeutics. Among the 146 supplemental indication approvals, 99 (68%) were for pharmacologics and 47 (32%) were for biologics; 74 (51%) were approved using at least 1 special regulatory program, including 20 using the Accelerated Approval pathway and 61 designated for Priority Review (Table 1). Most commonly, the supplemental indication approvals were for the treatment of cancer (78 approvals [53%]) or autoimmune and musculoskeletal (24 approvals [16%]) disease.

**Table 1.** Characteristics of Supplemental New Indication Drugs and Biologics Approved by the US FDA for New Indications from 2017 to 2019, and the New Drugs and Biologics Approved by the FDA for the Corresponding Original Indications

Characteristic	Approvals, No. (%)	
	Supplemental new indication (n = 146)^a^	Original (n = 109)
Approval year		
Before 2010	NA	23 (21)
2010-2014	NA	45 (41)
2015-2019	146 (100)	41 (38)
Agent type		
Pharmacologics	99 (68)	69 (63)
Biologics	47 (32)	40 (37)
Special regulatory program		
Any	74 (51)	57 (52)
Accelerated approval	20 (14)	25 (23)
Priority review	61 (42)	55 (50)
Fast track	NA	10 (9)
Breakthrough	35 (24)	17 (16)
Therapeutic area		
Autoimmune or musculoskeletal	24 (16)	5 (5)
Infectious diseases	5 (3)	6 (6)
Neurology	8 (5)	NA^b^
Dermatology	NA^b^	7 (6)
Cardiovascular, diabetes, and lipids	9 (6)	13 (12)
Cancer	78 (53)	46 (42)
Other	22 (15)	7 (6)

Abbreviations: FDA, US Food and Drug Administration; NA, not applicable.

^a^ Supplemental approvals are not eligible for Fast Track designation.

^b^ Therapeutic areas with fewer than 5 new indications or original indication approvals, respectively, are consolidated into the other category.

### Pivotal Trials Supporting Supplemental Indication Approvals

The FDA approved these 146 supplemental indications on the basis of 181 pivotal trials, with a median (interquartile range [IQR]) of 1 (1-1) trial per approval; 23 (15.8%) supplemental indication approvals were approved on the basis of at least 2 pivotal trials. Most pivotal trials supporting these indications used either an active (65 trials [35.9%; 95% CI, 29.3%-43.1%]) or placebo (77 trials [42.5%; 95% CI, 35.6%-49.8%]) comparator, and the majority of these multigroup trials were randomized (141 trials [99.3%; 95% CI, 96.0%-100.0%]) and double-blinded (106 trials [74.5%; 95% CI, 66.6%-81.0%]); 80 trials (44.2%; 95 CI, 37.2%-51.5%) used clinical outcomes as the primary efficacy end point (Table 2). The median (IQR) trial duration was 46.3 (16-117) weeks. The median (IQR) number of patients in the pivotal trials was 460 (226-734), and the median (IQR) number of patients in the intervention groups was 267 (149-473).

**Table 2.** Characteristics of Trials Supporting Supplemental New Indication Approvals by the US Food and Drug Administration Between 2017 and 2019

Characteristic	Trials, No.	Percentage (95% CI)						Trial duration, median (IQR), wk	Patients, median (IQR), No.	
		Randomized^a^	Double-blind^a^	Comparator			Clinical end point		Overall	Intervention group
				Placebo	Active	Single group				
Overall	181	99.3 (96.0-100.0)	74.6 (66.6-81.0)	42.5 (35.6-49.8)	35.9 (29.3-43.1)	21.6 (16.2-28.1)	44.2 (37.2-51.5)	46.3 (16-117)	460 (226-734)	267 (148.5-473)
Agent type										
Pharmacologic sNDAs	125	100.0 (96.4-100.0)	77.5 (68.4-84.5)	46.4 (37.9-55.1)	38.4 (30.3-47.2)	15.2 (9.9-22.5)	48.8 (42.5-59.8)	40.7 (12.2-121.3)	518 (276-797.5)	304 (158-480)
Biologic sBLAs	56	97.2 (85.5-99.5)	65.7 (49.2-79.2)	33.9 (22.9-47.0)	30.4 (19.9-43.3)	35.7 (24.5-48.8)	33.9 (22.9-47.0)	47.1 (24-101.7)	305.5 (163.8-559)	223.5 (125-338)
* P* value		.26	.18	.002	.002	.002	.08	.71	.02	.10
Special regulatory pathway										
Any	87	98.2 (90.2-99.7)	59.4 (46.0-71.3)	28.7 (20.3-39.0)	37.9 (28.5-48.4)	33.3 (24.3-43.8)	32.2 (23.3-42.6)	62.1 (30-128.6)	450 (207-726)	271 (149-444)
Accelerated approval	25	87.5 (52.9-97.8)	37.5 (13.7-69.4)	12.0 (4.2-30.0)	24.0 (11.5-43.4)	64.0 (44.5-79.8)	24.0 (11.5-43.4)	52.7 (34.1-107.4)	340 (177.5-743)	270 (152-424)
Priority review	74	97.9 (89.1-99.6)	64.6 (50.4-76.6)	33.8 (24-45.1)	36.5 (26.4-47.9)	29.7 (20.5-40.9)	32.4 (22.9-43.7)	56.4 (30-129)	449 (207.8-706.25)	256 (149.8-439.5)
Breakthrough	44	100 (85.1-100)	45.5 (26.9-65.3)	13.6 (6.4-26.7)	40.9 (27.7-55.6)	45.5 (31.7-59.9)	18.2 (9.5-31.9)	100.5 (42.1-128.9)	448.5 (206.2-710.8)	319.5 (159.5-319.5)
* P* value (any special regulatory program vs none)		.39	<.001	<.001	<.001	<.001	.02	.001	.004	.07
Therapeutic area										
Autoimmune or musculoskeletal	36	100 (89.3-100)	96.9 (84.3-99.4)	69.44 (53.1-82.0)	22.2 (11.7-38.1)	8.3 (2.9-21.8)	66.7 (50.3-79.8)	24 (14.5-25.7)	329 (188-546)	212.5 (104.3-344)
Cancer	91	98.3 (91.0-99.7)	50.8 (38.4-63.2)	23.1 (15.6-32.7)	41.8 (32.2-52.0)	35.2 (26.1-45.4)	27.5 (19.4-37.4)	94.9 (38.6-145.7)	501 (292-737)	297 (196-476)
Cardiovascular, diabetes, or lipids	11	100 (74.1-100)	100 (74.1-100)	54.6 (28.0-78.7)	45.5 (21.3-72)	0	81.8 (52.3-94.9)	100.3 (5.1-134.3)	4541 (2531-9341)	2266 (1252-4668)
Infectious diseases	6	100 (61.0-100)	66.7 (30.0-90.3)	16.7 (3.0-56.4)	83.3 (43.6-97.0)	0	66.7 (30.0-90.3)	3.8 (1-48)	621.5 (448-887.3)	311.5 (213.8-414.5)
Neurology	9	100 (67.6-100)	100 (67.5-100)	77.8 (45.3-93.7)	11.1 (2.0-43.5)	11.1 (2.0-43.5)	66.7 (35.4-87.9)	4 (4-6)	212 (146.5-375)	155 (89.5-251.5)
Other	28	100 (84.5-100)	85.7 (65.4-95.0)	57.1 (39.1-73.5)	21.4 (10.2-39.5)	21.4 (10.2-39.5)	42.9 (26.5-60.9)	18.5 (6-52)	291 (106.8-699.8)	199 (99-433.3)
* P* value (oncology v nononcology)		.43	<.001	<.001	<.001	<.001	<.001	<.001	.62	.88

Abbreviations: IQR, interquartile range; sBLA, supplemental biologics license application; sNDA, supplemental new drug application.

^a^ Excludes single-group studies.

### Supplemental Indication Approvals, Comparing Product Types

The 99 pharmacologic supplemental indication approvals were supported by 125 pivotal trials, whereas the 47 biologic approvals were supported by 56 trials (Table 2). The median (IQR) number of trials per approval was 1 (1-1) for pharmacologic approvals and 1 (1-1) for biologic approvals; 18 pharmacologic indications (18.2%) and 5 biologic indications (10.6%) were approved on the basis of at least 2 pivotal trials. Likewise, the trials supporting supplemental indication approvals were broadly similar in design between pharmacologics and biologics. Trials supporting pharmacologic approvals were as likely as trials supporting biologic approvals to be randomized (100.0% [95% CI, 96.4%-100.0%] vs 97.2% [95% CI, 85.5%-99.5%]; *P* = .26) and double-blind (77.5% [95% CI, 68.4%-84.5%] vs 65.7% [95% CI, 49.2%-79.2%]; *P* = .18) when multigrouped (106 trials for pharmacologic approvals and 36 trials for biologic approvals), and to use a clinical outcome as their primary efficacy end point (48.8% [95% CI, 42.5%-59.8%] vs 33.9% [95% CI, 22.9%-47.0%]; *P* = .08), but were more likely to have either an active or placebo comparator group (84.8% [95% CI, 77.5%-90.9%] vs 64.3% [95% CI, 51.2%-75.5%]; *P* = .002). The duration of trials supporting pharmacologic approvals did not differ from the duration of trials supporting biologic approvals (median [IQR], 40.7 [12.2-121.3] vs 47.1 [24-101.7] weeks). The number of overall patients in the pivotal trials (median [IQR] 518 [276-797.5] vs 305.5 [163.8-559] patients) and in the intervention groups (median [IQR], 304 [158-480] vs 223.5 [125-338] patients) did not differ between pharmacologic and biologic approvals.

When aggregating all the pivotal trials that supported the indication approval, the number of patients overall (median [IQR], 514 [269-834] vs 306 [157-713] patients) and in the intervention groups overall (median [IQR], 281.5 [155-451] vs 222 [105-340] patients) did not differ between pharmacologic and biologic approvals (Table 3). Pharmacologic and biologic approvals did not differ in there being at least 1 pivotal trial that was randomized (81.8% [95% CI, 73.1%-88.2%] vs 63.8% [95% CI, 49.5%-76.0%]; *P* = .02), double-blinded (65.7% [95% CI, 55.9%-74.3%] vs 44.7% [95% CI, 31.4%-58.8%]; *P* = .02), used a clinical outcome as the primary efficacy end point (51.5% [95% CI, 41.8%-61.1%] vs 31.9% [95% CI, 20.4%-46.2%]; *P* = .03), and had a duration of at least 6 months (66.7% [95% CI, 56.9%-75.2%] vs 72.3% [95% CI, 58.2%-83.1%]; *P* = .49) or 12 months (54.6% [95% CI, 44.8%-64.0%] vs 55.3% [95% CI, 41.2%-68.6%]; *P* = .93), but pharmacologic approvals were more likely to have at least 1 pivotal trial that used either an active or placebo comparator (84.8% [95% CI, 76.5%-90.6%] vs 66.0% [95% CI, 51.7%-77.8%]; *P* = .009) (Table 4).

**Table 3.** Number and Characteristics of Aggregated Pivotal Efficacy Trials Providing the Basis for Original and Supplemental Indication Approvals by the US Food and Drug Administration Between 2017 and 2019, Stratified by Supplemental or Original Indication, Therapeutic Agent, and Indication Characteristics

Variable	Trials, No.	Pivotal efficacy trials, median (IQR), No.	Patients in aggregated pivotal efficacy trials, median (IQR), No.	
			Overall	Intervention group
Supplemental or original indication				
Original	109	1 (1-2)	326 (161.5-676)	256.3 (120.1-375.4)
Supplemental	146	1 (1-1)	445 (211.8-799.3)	256.5 (144.8-447.3)
* P* value		<.001	.05	.12
Therapeutic agent				
Pharmacologic sNDA	99	1 (1-1)	514 (269-834)	281.5 (155-451)
Biologic sBLA	47	1 (1-1)	306 (157-713)	222 (105-340)
* P* value		.26	.05	.16
Therapeutic area				
Oncology sNDA or sBLA	78	1 (1-1)	505 (300.8-873)	286 (200-471.5)
Nononcology sNDA or sBLA	68	1 (1-1)	334.3 (153.3-749.8)	215.5 (99-421)
* P* value		.13	.06	.04

Abbreviations: IQR, interquartile range; sBLA, supplemental biologics license applications; sNDA, supplemental new drug application.

**Table 4.** Proportion of Indication-Level Approvals of Novel Therapeutic Agents by the US Food and Drug Administration on the Basis of at Least 1 Trial That Met the Criteria Below, Stratified by Supplemental or Original Indication, Therapeutic Agent, and Indication Characteristics

Variable	Trials, No.	Percentage (95% CI)						
		≥2 Pivotal trials	≥1 Randomized trial	≥1 Double-blind trial	Trial duration		≥1 Trial	
					≥6 mo	≥12 mo	With active or placebo comparator	With clinical outcome as primary efficacy end point
Supplemental or original indication								
Original	109	44.0 (33.7-42.6)	80.0 (71.4-86.5)	63.8 (54.3-72.4)	51.5 (43.3-62.6)	27.6 (17.9-35.0)	78.8 (70.0-85.6)	53.9 (46.2-65.0)
Supplemental	146	15.8, (10.7-22.5)	76.0 (68.5-82.2)	58.9 (50.8-66.6)	68.5 (60.6-75.5)	54.8 (46.7-62.6)	78.9 (71.4-84.6)	45.2 (37.4-53.3)
* P* value		<.001	.54	.51	.01	<.001	.87	.18
Therapeutic area								
Pharmacologic sNDA	99	18.2 (11.8-26.9)	81.8 (73.1-88.2)	65.7 (55.9-74.3)	66.7 (56.9-75.2)	54.6 (44.8-64.0)	84.8 (76.5-90.6)	51.5 (41.8-61.1)
Biologic sBLA	47	10.6 (4.6-22.6)	63.8 (49.5-76.0)	44.7 (31.4-58.8)	72.3 (58.2-83.1)	55.3 (41.2-68.6)	66.0 (51.7-77.8)	31.9 (20.4-46.2)
* P* value		.24	.02	.02	.49	.93	.009	.03
Therapeutic area								
Oncology sNDA or sBLA	78	11.5 (6.2-20.5)	68.8 (57.9-77.8)	37.5 (27.7-48.5)	93.6 (85.9-97.2)	76.9 (66.4-84.9)	71.9 (61-80.6)	32.1 (22.7-43.0)
Nononcology sNDA or sBLA	68	20.6 (12.7-31.6)	84.8 (74.3-91.6)	84.8 (74.3-91.6)	39.7 (28.9-51.6)	29.4 (19.9-41.1)	86.8 (76.7-92.9)	60.3 (48.4-71.1)
* P* value		.10	.04	<.001	<.001	<.001	.03	<.001

Abbreviations: sBLA, supplemental biologics license application; sNDA, supplemental new drug application.

### Supplemental Indication Approvals, Comparing Therapeutic Areas

The 78 oncology supplemental indication approvals were supported by 91 pivotal trials, whereas the 68 nononcology approvals were supported by 90 pivotal trials (Table 2). The median (IQR) number of trials per approval was 1 (1-1) and 1 (1-1), respectively, and 9 oncology indications (11.5%) and 14 nononcology indications (20.6%) were approved on the basis of at least 2 pivotal trials (difference, 9.1%; 95% CI, 2.9%-21.0%; *P* = .10). The trials supporting therapeutics used for oncology indications did not differ significantly from the trials supporting indications used for all other therapeutic areas with respect to use of randomization (98.3% vs 100.0%; difference, 1.7%; 95% CI, 1.6%-5.0%; *P* = .43), but were significantly less likely to be double-blinded (50.8% vs 92.3%; difference, 41.5%; 95% CI, 27.4%-55.5%; *P* < .001) when multigrouped (59 oncology trials and 78 nononcology trials); overall, these trials were less likely to use either a placebo or active comparator group (64.9% vs 86.7%; difference, 21.8%; 95% CI, 9.8%-33.9%; *P* < .001) and to use clinical outcomes as their primary efficacy end point (27.5% vs 61.1%; difference, 33.6%; 95% CI, 14.1%-40.9%; *P* < .001). However, trials supporting oncology indications were longer than trials supporting indications in all other therapeutic areas (median [IQR], 95 [39-146] weeks vs 17 [6-48] weeks). The number of overall patients the pivotal trials (median [IQR], 501 [292-737] vs 393.5 [203-747] patients) and in the intervention groups (median [IQR], 297 [196-476] vs 243 [104.8-449.3] patients) did not differ between trials supporting oncology indications and indications in all other therapeutic areas.

When aggregating all the pivotal trials that supported the indication approval, the number of patients overall (median [IQR], 505 [300.8-873] vs 334.3 [153.3-749.8] patients) and in the intervention groups (median [IQR], 286 [200-471.5] vs 215.5 [99-421] patients) did not differ between oncology and nononcology approvals (Table 3). Oncology approvals were not less likely to be supported by at least 1 pivotal trial that was randomized (68.8% [95% CI, 57.9%-77.8%] vs 84.8% [95% CI, 74.3%-91.6%]; *P* = .04) or used either an active or placebo comparator (71.9% [95% CI, 61.0%-80.6%] vs 86.8% [95% CI, 76.7%-92.9%]; *P* = .03), but were less likely to be supported by at least 1 trial that was double-blinded (37.5% [95% CI, 27.7%-48.5%] vs 84.8% [95% CI, 74.3%-91.6%]; difference, 47.3%; 95% CI, 33.9%-61.3%; *P* < .001) and that used a clinical outcome as the primary efficacy end point (32.1% [95% CI, 22.7%-43.0%] vs 60.3% [95% CI, 48.4%-71.1%]; difference, 28.2%; 95% CI, 12.7%-43.8%; *P* < .001), and were more likely to be supported by at least 1 trial that had a duration of at least 6 months (93.6% [95% CI, 85.9%-97.2%] vs 39.7% [95% CI, 28.9%-51.6%]; difference, 53.9%; 95% CI, 41.0%-66.7%; *P* < .001) or 12 months (76.9% [95% CI, 66.4%-84.9%] vs 29.4% [95% CI, 19.9%-41.1%]; difference, 47.5%; 95% CI, 33.2%-61.8%; *P* < .001) (Table 4).

### Supplemental Indication Approvals Compared With Original Indication Approvals

The 107 therapeutics for which we identified supplemental indication approvals were originally approved by the FDA for 109 clinical indications (Table 1); original indication approvals took place between 1991 and 2018, and 18% (19 of 107 approvals) were approved for a supplemental indication in a different therapeutic area from the original indication approval, the majority of which (11 approvals) were approved for a supplemental indication for use for autoimmune or musculoskeletal disease after an original approval for a different clinical use. The median (IQR) number of trials per approval was 1 (1-2) and 1 (1-1), respectively (Table 3); original indication approvals were significantly more likely to be supported by at least 2 pivotal efficacy trials compared with supplemental indication approvals (44.0% [95% CI, 33.7%-42.6%] vs 15.8% [10.7%-22.5%]; difference, 28.2%; 95% CI, 17.6%-39.6%; *P* < .001). There was no difference between original and supplemental indication approvals with respect to being supported by at least 1 pivotal trial that was randomized (80.0% [95% CI, 71.4%-86.5%] vs 76.0% [95% CI, 68.5%-82.2%]; *P* = .54), double-blinded (63.8% [95% CI, 54.3%-72.4%] vs 58.9% [95% CI, 50.8%-66.6%]; *P* = .51), used either an active or placebo comparator (78.8% [95% CI, 70.0%-85.6%] vs 78.9% [95% CI, 71.4%-84.6%]; *P* = .87), and used a clinical outcome as the primary efficacy end point (53.9% [95% CI, 46.2%-65.0%] vs 45.2% [95% CI, 37.4%-53.3%]; *P* = .18); however, original indication approvals were significantly less likely to be supported by at least 1 trial that had a duration of at least 6 months (51.5% [95% CI, 43.3%-62.6%] vs 68.5% [95% CI, 60.6%-75.5%]; difference, 17.0%; 95% CI, 3.7%-27.8%; *P* = .01) or 12 months (27.6% [95% CI, 17.9%-35.0%] vs 54.8% [95% CI, 46.7%-62.6%]; difference, 27.2%; 95% CI, 14.5%-37.8%; *P* < .001) (Table 4). There was no difference between original and supplemental indication approvals with respect to the number of patients in the aggregated pivotal trials overall (median [IQR], 326 [62-676] vs 445 [162-676] patients) or the number in the intervention groups (median [IQR], 256 [120-375] vs 257 [145-447] patients).

## Discussion

Among all supplemental new indications approved by the FDA between 2017 and 2019, we found that the number and design of the pivotal trials supporting supplemental indication approvals varied on the basis of therapeutic area, with the strength of evidence for cancer indications weaker than those for other indications. However, there was little difference in the number and design of the pivotal trials supporting pharmacologic and biologic supplemental indication approvals, or between supplemental and original indication approvals by the FDA. These findings suggest that the evidentiary standards for supplemental new indication approvals varies across therapeutic areas but that the FDA’s standard for approval is consistent between supplemental and original indications for use.

Our study suggests that the trials supporting supplemental indication approvals for oncology therapeutics vary significantly from those trials supporting indications in all other therapeutic areas, with a tendency to be fewer in number and less robust in design compared with trials supporting supplemental approvals for indications in all other therapeutic areas. This variation highlights the flexible approach taken by the FDA, guided by the severity of the disease and availability of treatment alternatives. Flexible standards, with greater laxity for oncology approvals, was previously demonstrated in our study^2^ characterizing the pivotal efficacy trials supporting original indication approval of novel therapeutics. In addition, substantial variation has been described with regard to the pivotal efficacy trials supporting the approval of novel oncology therapeutics.^16^

The evidentiary standard for supplemental new indication approval has important implications for clinical care and regulatory decision-making, offering insight into whether the FDA follows the precedent set by the therapeutic’s original approval or whether a lower, or even a higher, standard is used. There are competing reasons for why the FDA might adopt a different standard for supplemental approvals. On the one hand, if safety was established through the agency’s original approval, some might argue that additional trials are not needed for other clinical uses. Instead, safety may be inferred from prior use. This scenario plays out frequently through off-label prescribing of approved drugs and biologics, as reported by 21% of office-based physicians^17^ and as occurs among 80% of inpatient episodes of care among certain subpopulations, such as children.^18^ As opposed to physicians prescribing therapies off-label, with little to no evidence, perhaps regulatory standards could be lowered to incentivize any postmarket clinical evidence generation for new clinical uses after original approval.^19^

On the other hand, there is an argument that standards could be strengthened once a therapeutic is available for widespread use. The FDA could require larger and longer duration trials to more fully characterize longer-term risks of approved therapeutics, and this higher standard approach could preclude the use of surrogate markers as primary trial end points. Thus, the FDA could require more robust clinical trial designs to demonstrate drug safety and efficacy that would better inform physician-patient decision-making and coverage decisions. For more expensive therapies, supplemental new indication approval by the FDA generally guarantees insurance coverage for that therapeutic use, as opposed to broad coverage of the therapeutic for any use. For instance, the Centers for Medicare & Medicaid Services provides coverage of oncology drugs for any FDA-approved indication for use, as well as any off-label use listed in certain compendia, facilitating patient access even while supplemental indications are tested by sponsors and evaluated by the FDA.

Our findings suggest that the FDA maintains consistent evidentiary standards for both supplemental new and original indication approvals. However, ongoing efforts to increasingly leverage observational studies to support regulatory evaluations and approvals, as opposed to clinical trials, may affect these standards. The 21st Century Cures Act, which was signed into law on December 13, 2016, contains a provision to promote the use of real-world evidence (RWE)—that is, studies of drugs and biologics that are derived from data collected in routine medical settings. In response, the FDA launched an initiative to explain the agency’s former use of RWE for safety evaluations and plans for future uses,^20^ including for supplemental new indication approvals.^21^ Even before this legislation, the FDA had used RWE to inform approvals, such as in 2014, when blinatumomab (Blincyto) received accelerated approval by the FDA for the treatment of relapsed or refractory, Philadelphia chromosome–negative, acute lymphoblastic leukemia on the basis of a single-group, open-label phase 2 study. In this case, RWE came in the form of historical controls who received standard of care, and efficacy was shown according to a weighted analysis of data from medical record reviews.^22^

Generating additional drug safety and effectiveness evidence through observational research may offer advantages with respect to time and resources, compared with clinical trials. However, there are outstanding issues to resolve, including data definitions, establishing which data are fit for purpose, and establishing best practices,^23^ such as study registration.^24^ Furthermore, our recent study^25^ found that few of the recently published, highest-impact trials could be feasibly replicated using RWE because much of the data needed for randomized clinical trials are not captured in electronic health records in a structured format, if at all. Although the strengths and limitations of using RWE for the evaluation of supplemental new indication approvals for therapeutics already available for use are better characterized, there is a clear opportunity to complement randomized clinical trials to offer more information on product safety and efficacy.

### Limitations

Our study has some limitations. First, we only assessed a recent, 3-year sample of therapeutics approved by the FDA for supplemental new clinical indications and, therefore, could not capture long-term trends in supplemental indication approvals or supplemental indication approvals by other national regulators. Second, our study did not evaluate whether the FDA considered other data regarding use of these therapeutics in other countries, which may have informed the agency’s efforts to evaluate supplemental new indication approval safety and efficacy.

## Conclusions

In this cross-sectional study, we found that there was little difference in the evidence supporting supplemental and original indication approvals by the FDA, but the number and design of pivotal trials supporting supplemental indication approvals varied according to therapeutic area, with the strength of evidence for cancer indications weaker than that for other indications. Overall, our results highlight the need for ongoing postmarket evaluation of supplemental new indications to ensure our understanding of product benefit and safety, as well as to inform both clinicians’ and policy makers’ decisions, including ongoing efforts to leverage observational studies and RWE, as opposed to clinical trials, to support FDA regulatory decisions and evaluations.
